# Higher cMET dependence of sacral compared to clival chordoma cells: contributing to a better understanding of cMET in chordoma

**DOI:** 10.1038/s41598-021-92018-0

**Published:** 2021-06-14

**Authors:** Birgit Lohberger, Susanne Scheipl, Ellen Heitzer, Franz Quehenberger, Danielle de Jong, Karoly Szuhai, Bernadette Liegl-Atzwanger, Beate Rinner

**Affiliations:** 1 Department of Orthopedics and Trauma, Medical University Graz, Auenbruggerplatz 5-7, 8036 Graz, Austria; 2 Diagnostic and Research Institute of Human Genetics, Medical University of Graz, Graz, Austria; 3 Institute for Medical Informatics, Statistics and Documentation, Medical University of Graz, Graz, Austria; 4 Department of Molecular Cell Biology, Leiden University Medical Center, Leiden, The Netherlands; 5 Diagnostic and Research Institute of Pathology, Medical University Graz, Graz, Austria; 6 Division of Biomedical Research, Medical University Graz, Graz, Austria

**Keywords:** Cancer, Cell biology, Medical research

## Abstract

Chordomas are rare slow growing, malignant bone tumors of the axial skeleton with no approved medical treatment. As the majority of chordomas express cMET and its ligand, HGF, and crosstalks between EGFR and MET-signaling exist, we aimed to explore cMET activity in chordoma cell lines and clinical samples. We investigated nine chordoma patients and four chordoma cell lines for cMET expression. Two clival and two sacral chordoma cell lines were tested for chromosomal abnormalities of the *MET* gene locus; we studied the influence of HGF on the autocrine secretion and migration behavior, as well as protein expression and phosphorylation. Two MET/ALK inhibitors were investigated for their effects on cell viability, cell cycle, cyclin alterations, apoptosis, and downstream signaling pathways. Moderate and strong expression of membrane and cytoplasmic cMET in chordoma patients and cell lines used, as well as concentration-dependent increase in phospho cMET expression after HGF stimulation in all four chordoma cell lines was shown. U-CH2, MUG-Chor1, and UM-Chor1 are polysomic for *MET.* Chordoma cell lines secreted EGF, VEGF, IL-6, and MMP9 upon HGF-stimulation. Sacral cell lines showed a distinct HGF-induced migration. Both inhibitors dose-dependently inhibited cell growth, induce apoptosis and cell-cycle arrest, and suppress downstream pathways. Heterogeneous responses obtained in our in vitro setting indicate that cMET inhibitors alone or in combination with other drugs might particularly benefit patients with sacral chordomas.

## Introduction

Chordomas are rare, malignant, locally destructive, slowly growing bone tumors that show an invasive growth behavior; prognosis is poor with a median survival of seven years after diagnosis. Current treatment concepts include surgical en-bloc resection with negative margins and high-dose radiation therapy as well as chemotherapy for the extremely rare dedifferentiated chordomas^1,2^. Several authors have suggested an interaction between hepatocyte growth factor (HGF)/cMET and epidermal growth factor receptor (EGFR) signalling^3,4^. Although chordomas have a very heterogeneous geno- and phenotype, gains can be observed on chromosome 7q31.2, the site of the cMET receptor^5^. cMET is activated upon binding of its sole ligand HGF, which is either released by fibroblasts within the tumor-microenvironment or by tumor-cells themselves in an autocrine manner^6^. MET-signaling regulates cell survival, migration, and proliferation^7^. Changes in cMET expression levels due to mutations and amplifications comprise well-known resistance mechanisms in other types of cancer^8^. Strong HGF and MET expression has been reported in a majority of clinical chordoma samples^9,10^.

The present study aims to further investigate the role of cMET in chordomas in vitro: two sacral (MUG-Chor1, U-CH2) and two clival (MUG-CC1, UM-Chor1) chordoma cell lines were karyotyped and then tested in functional studies. To mimic physiological conditions, we stimulated tumor cells with HGF. Protein expression, phosphorylation, cytokine secretion and migration potential were analyzed. Furthermore, the effects of the MET/ALK inhibitors crizotinib and cabozantinib were investigated in terms of viability assessment, cell cycle arrest, cyclin alterations, apoptosis and analyses of downstream signaling pathways. These studies contribute to a better understanding of cMET and HGF in chordoma biology.

## Results

### Immunohistochemical evaluation of cMET expression in chordoma patients

**Figure 1:**
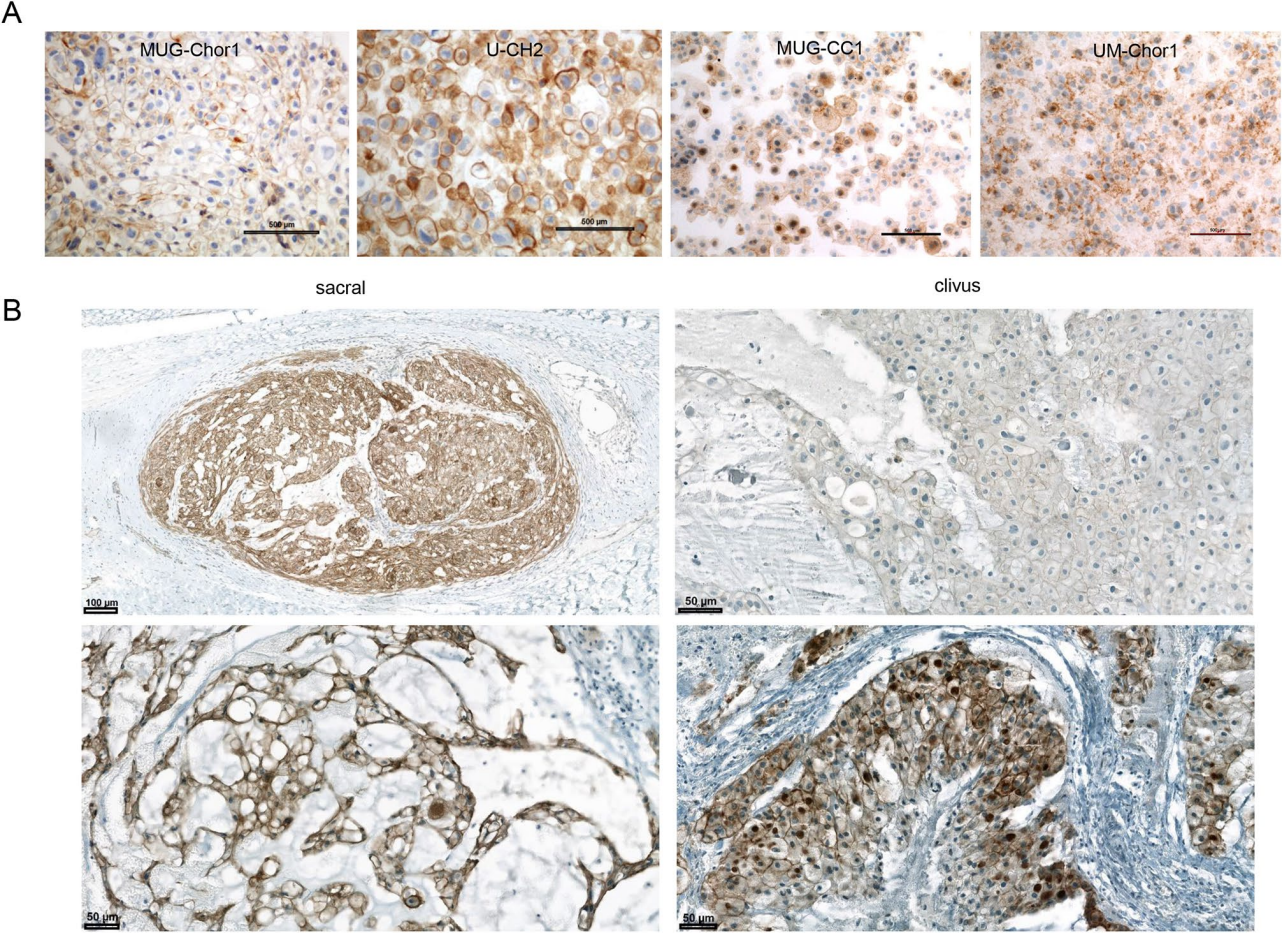
cMET immunohistochemical analyses. (**A**) Representative cMET immunohistochemical analyses from chordoma cell lines MUG-Chor1, U-CH2, MUG-CC1, and UM-Chor 1. Every cell line presented a high cMET expression; (**B**) Representative cMET immunohistochemical analysis from clival and sacral chordoma patient.

To figure out the cMET expression, immunohistochemical staining was performed on different chordoma cell lines as well as on patient samples. All four chordoma cell lines showed a moderate or strong expression of membrane and cytoplasmic cMET immunostaining (Fig. 1A). cMET was also detected in all patient samples (5 sacral, 4 clival). Staining intensity varied from moderate to strong. Due to the small number of samples, no significant differences in cMET expression was found between clival and sacral samples (Fig. 1B).

### Karyotyping and MET amplification

**Figure 2:**
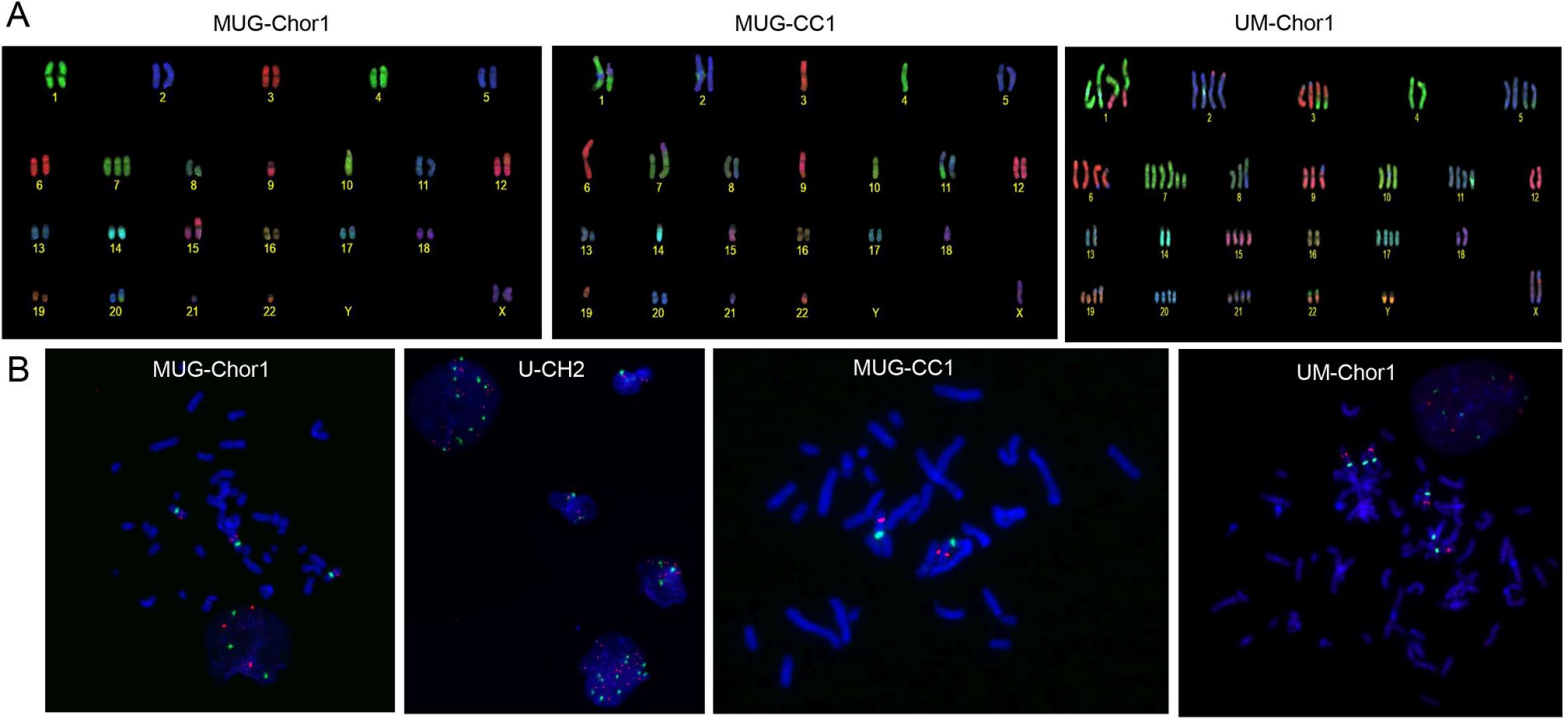
Karyotyping and *MET* amplification. (**A**) The resulting karyotpe of MUG-Chor1 was 42-43,<2n>,XX,+7,der(9;15),der(10)hsr(q?),10,der(12)t(12;19)(p;q)hsr(19)(q?), der(17,21)(q10;q10),der(20)t(10;20),-18,-19,-22. Karyograms showed a hypodiploid genome with variable translocations; these most commonly involved chromosomes 4, 14, 18, 20. The karyoptype of MUG-CC1 was 33-34,<n+>,X,Y,del(1)(q2?),+der(1;18)(q;qter),+2,+5,der(6)t(6;6)(p;q),der(7)t(X;16)(q;q)t(7;16)(p;q),der(8)t(8;11)(q;p),der(9)t(9;19)(q;q),der(11)t8;11)(q;p)t(11;13)(q;q),+der(11)t(1;11)(q;q),+12,der(13)t(11;13)(p;q),+del(16)(q),+del(17)(p),+20^23^. Hyperhaploid karyotype with translocations that involved the chromosome X fragment to form a dicentric chromosome with chromosome 7 or 8 and a long arm of chromosome 17 attached to chromosome 8 or 11 or to derivative chromosome 11. The karyoptype of UM-Chor1 was 71-77,<3n+>XYY, +der(X),der(1;12),+1,t(2;6)(p;q)x2,+1,der(3)t(3;20)(q;q),der(3),4,t(5;8)(q;q)x2,+5,+6,+7,+del(7)(q)x2,der(9),12,13,14,+15,16,+17,18,ider(18)(q),der(19)t(19;13),+19,+20,+21,der(21)t(5;21),der(22)t(4;22),-22. It showed a hypertriploid karyotype with complex rearranged chromosomes 1 and 3 and variable partners involving the X chromosome. (**B**) Representation of MET locus in the metaphases of all four chordoma cell lines.

**Figure 3:**
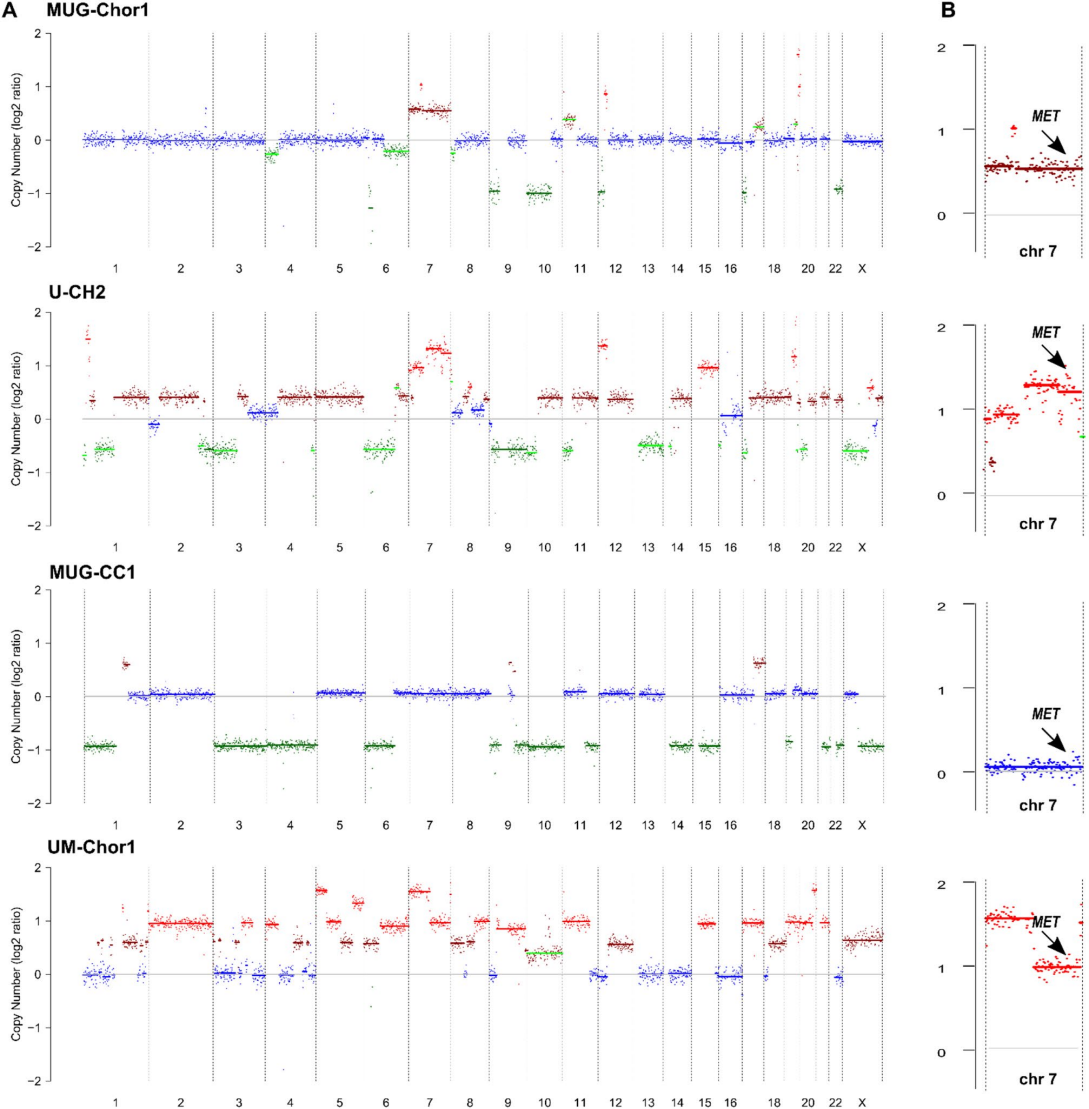
CNVs and cMET amplification. (**A**) Shown are genome-wide copy number profiles established with the ichorCNA algorithm. Plotted are log2-ration of read counts from 1 Mb bins. Blue indicated balanced region, green indicates losses of chromosomal material, red and brown indicated gain of chromosomal material. The profile of MUG-CC1 has been adjusted scoring to the FISH karyotyping results. (**B**) Copy number status of chromosome 7. Arrows indicate location of the *MET* gene.

Molecular karyotyping of the chordoma cell lines was carried out using a multicolor-Fluorescence in situ hybridisation (FISH) technique (Fig. 2A). While MUG-Chor1 and MUG-CC1 presented a hypodiploid and hyperhaploid genome with variable translocations, respectively, UM-Chor1 showed a hypertriploid karyotype with complex rearranged chromosomes. Detailed karyotypes are shown in Fig. 2. Genomewide copy number profiling and ploidy estimates established from shallow whole genome sequencing (sWGS) were highly concordant (Fig. 3). cMET status was assessed using multicolour FISH and from sWGS data. For MUG-Chor1 a total of 3 *MET* locus specific and centromeric FISH signal were detected, which confirmed the polysomy 7 from karyotyping and sWGS (Fig. 2B). In U-CH2 cells the combination of chromosome 7 centromeric probes and the *MET* locus specific FISH in metaphases showed three centromeric signals and 4 *MET* specific signal cluster from which two were with stronger signals indicating a possible amplification of the region. In interphase different levels of polyploidization were observed resulting between 3 and 11 centromeric signals and 6–19 *MET* locus specific signals between the different cells. This was in line with the sWGS sequencing data, which identified a focal amplification on an already gained chr7q. MUG-CC1 showed no *MET* amplification, and the combination of chromosome 7 centromeric probes and the *MET* locus specific FISH showed the presence of two copies of each signals. In accordance with the karyotyping results, one signal was unchanged, but the second was rearranged on chromosome 7. Interestingly, focal amplification calling indicated an amplification of *MET* for UM-Chor1 that was not confirmed by FISH. UM-Chor1 cell line showed the presence of 6 copies of chromosome 7 centromeric signals and a consistent 4 copies of MET locus specific signals in line with the karyotyping results (Table 1).

**Table 1:** Amplification MET and ALK.

NM_001127500.2	*MET*	chr7:116312444–116438440		7q31.2			
Sample	Chr	Start	End	Size [Mb]	log2	Hypothetical copy number	Copy number status
MUG-Chor1	chr7	100503600	124968820	24.47	0.39	2.63	Gain
MUG-CC1	chr7	115750949	116938451	1.19	0.61	3.04	Gain
UM-Chor1	chr7	116266181	116481209	0.22	2.41	10.64	Amplification
U-CH2	chr7	116266181	116481209	0.22	3.29	19.55	Amplification

NM_004304.4	*ALK*	chr2:29415640–30144477		2p23.2–2p23.1			
Sample	Chr	Start	End	Size [Mb]	log2	Hypothetical copy number	Copy number status
MUG-Chor1	chr2	21304337	87366690	66.06	− 0.17	1.77	Loss
MUG-CC1	chr2	23169294	32270013	9.10	0.64	3.11	Gain
UM-Chor1	chr2	29384280	29496976	0.11	0.89	3.70	Gain
		29496977	54377577	24.88	0.49	2.81	Gain
U-CH2	chr2	29384280	29496976	0.11	1.63	6.20	Gain
		29496977	40528426	11.03	− 0.06	1.92	Gain

### Hepatocyte growth factor (HGF) stimulates cMET signalling in chordoma cells

**Figure 4:**
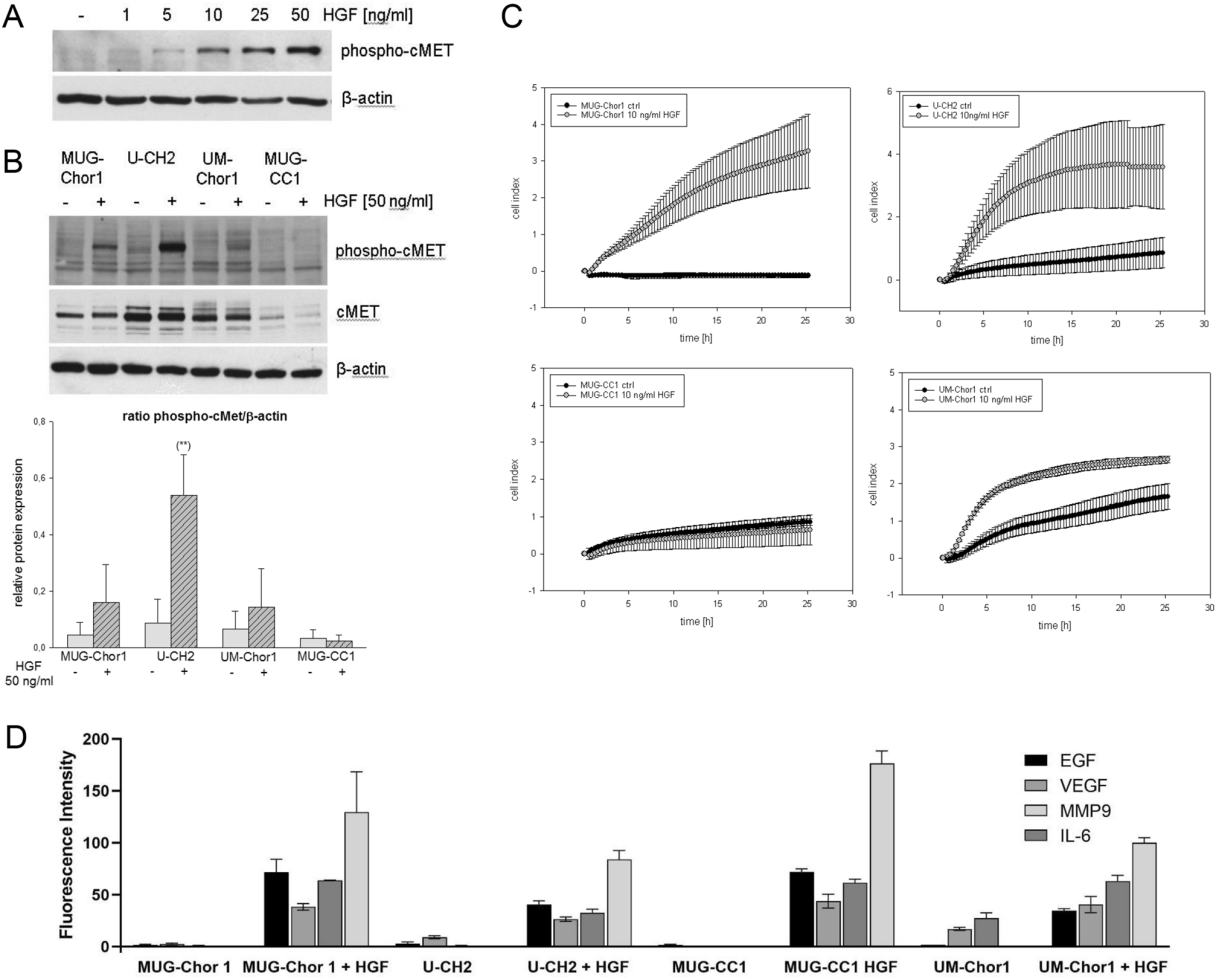
cMET phosphorylation and migration analysis after HGF stimulation. (**A**) The concentration depending increase of phospho-cMET expression after the treatment with 0, 1, 5, 10, 25 and 50 ng/ml HGF; (**B**) Protein expression of cMET and phospho-cMET after 50 ng/ml HGF treatment in all four chordoma cell lines. One representative blot out of three is shown. β-actin was used as loading control. (**C**) Effect of HGF on migration of chordoma cell lines. Chordoma cells were plated onto the upper well of migration plate, and HGF was added to the lower chamber at 50 ng/ml or culture medium alone (ctrl). The cells on the lower side of the membrane were counted after 24 h (mean ± SD, n = 3). (**D**) After the stimulation with 50 ng/ml HGF for 60 min, cell culture supernatants were harvested and EGF, VEGF, IL-6, and MMP9 were measured by the xMAP technology. The mean fluorescence intensity (MFI) was presented.

To determine the appropriate concentration for HGF stimulation, MUG-Chor1 cell line was treated with 0, 1, 5, 10, 25, and 50 ng/ml HGF. A concentration dependent increase of phospho-cMET expression was observed (Fig. 4A). Based on preliminary data, cells were stimulated in all subsequent experiments with 50 ng/ml HGF for 60 min. We analysed the protein expression of cMET and phospho-cMET under HGF stimulation in all four chordoma cell lines by western blot analysis (Fig. 4B). A ratio of phospho-cMET versus β-actin was calculated (mean ± SD; n = 3). Highest cMET expression and phosphorylation was seen in U-CH2 (-HGF: 0.1 ± 0.1 vs + HGF: 0.54 ± 0.2; p = 0.006), followed by MUG-Chor1 (-HGF: 0.05 ± 0.02 vs + HGF: 0.16 ± 0.2) and UM-Chor1 (-HGF: 0.08 ± 0.05 vs + HGF: 0.18 ± 0.05). MUG-CC1 showed the lowest cMET signal (-HGF: 0.03 ± 0.02 vs + HGF: 0.02 ± 0.01) compared to the other tested chordoma cell lines.

### HGF effect on chordoma cell migration correlates with cMET protein levels

To assess the role of HGF on chordoma cell migration, we studied the cell lines´ migration behaviour upon stimulation with HGF using the real-time xCELLigence system. We found that HGF distinctly enhanced the migration of two sacral chordoma cell lines MUG-Chor1 and U-CH2 cells, moderately attracted the clival UM-Chor1 cells, and did not affect MUG-CC1 cells (Fig. 4C).

Stimulation with HGF leads to secretion of the epidermal growth factor (EGF), the vascular endothelial growth factor (VEGF), interleukin-6 (IL-6), and the matrix metalloproteinase 9 (MMP9).

To visualize the HGF/cMET axis in chordoma cells, all four chordoma lines were stimulated with HGF and the expression of angiogenesis modulating enzymes such as VEGF and MMP9, pro-inflammatory IL-6, and EGF, the synergistic growth factor to HGF, was measured by multiplex analysis. Samples were analysed in duplicates and related to the cell amount used. Data were presented in the mean fluorescence intensity (MFI) (Fig. 4D). MUG-Chor1, U-CH2, and MUG-CC1 treated with HGF showed an intense increase of EGF, VEGF, IL-6, and MMP9 compared to non-treated control cells. UM-Chor1 cells showed the least increase in measured cytokines compared to the control and the other chordoma cell lines. The expression of the cytokines was used to functionally demonstrate that the cMET/HGF axis is active in all chordoma cells. In all four chordoma cell lines, changes in expression were most distinct for MMP9.

### Influence of crizotinib and cabozantinib on cell viability

**Figure 5:**
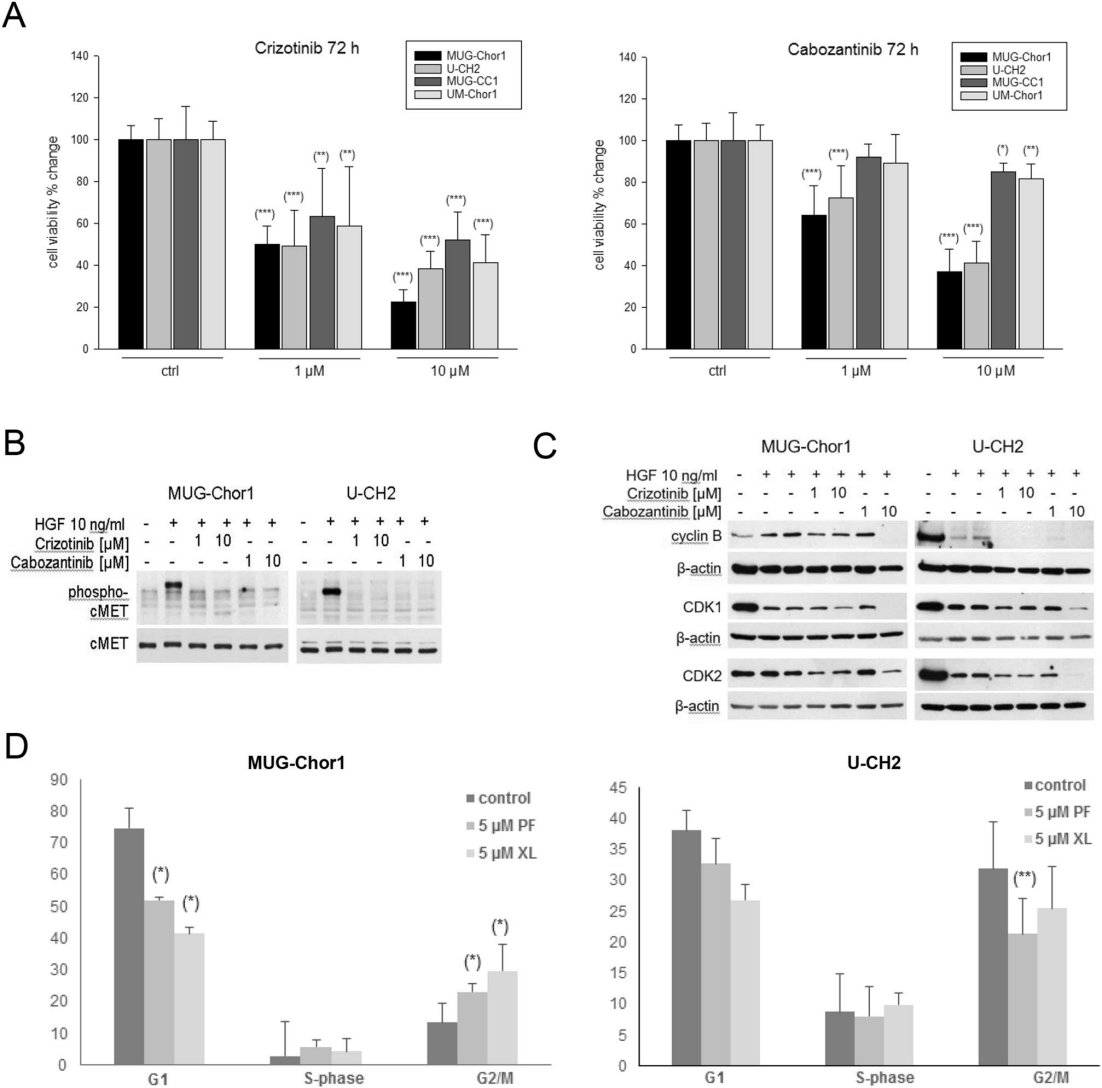
Influence of the cMET inhibitors crizotinib and cabozantinib on viability and cell cycle. (**A**) Influence of 1 and 10 µM crizotinib and cabozantinib on the viability of HGF stimulated chordoma cells after 72 h. Results were normalized to untreated controls (+GF) and represent mean ± SD of at least three independent experiments done in quadruplicates. (**B**) Protein expression of cMET and phospho-cMET under the influence of 1 and 10 µM crizotinib and cabozantinib. (**C**) Protein expression of the G2/M checkpoint proteins cyclin B, CDK1 and CDK2 were significantly reduced according to the G2/M arrest. (**D**) Data derived from FACS cell cycle measurements showed a decrease in the number of cells in G1- and S phase, which was accompanied by a pronounced increase of cells in G2/M phase in both sacral cell lines MUG Chor1 and U-CH2. Cell populations are given as percentage of total cells (mean of n = 3).

Stimulation with 10 ng/ml HGF for 1 h lead to a significant increase of cell viability in chordoma cells after 72 h. Subsequent treatment with crizotinib for 72 h decreased the viability of all four chordoma cell lines highly significant in a concentration dependent manner (Fig. 5A, left). IC_50_ values after crizotinib treatment were 189 nM MUG-Chor1, 314 nM U-CH2, 442 nM MUG-CC1, and 663 nM UM-Chor1, respectively.

Cabozantinib reduced viability in the two sacral chordoma cell lines MUG-Chor1 and U-CH2 significantly more efficiently than in the two clival cell lines MUG-CC1 and UM-Chor. IC_50_ values after cabozantinib treatment were 433 nM MUG-Chor1 and > 10 µM for the other three cell lines. Due to the minor effects it was not possible to fit the IC_50_ values. (Fig. 5A, right). For evaluation of the phospho-cMET levels, we used MUG-Chor1 and U-CH2, as these were most affected by the previous treatments. Phospho-cMET levels were significantly decreased upon treatment with crizotinib and cabozantinib, indicating that these drugs actually acted as MET-inhibitors (Fig. 5B).

### MET-inhibitors induce cell-cycle changes in chordoma cell lines

Treatment of chordoma cells with 5 µM crizotinib (PF) and cabozantinib (XL), respectively, resulted in a cell cycle shift. Both inhibitors led to a decrease in cells in the G1 phase and an associated G2 arrest. The protein expression of the corresponding G2/M checkpoint proteins cyclin B, CDK1, and CDK2 were significantly decreased (Fig. 5C). Flow cytometry analysis revealed that the number of cells in the G1 phase was reduced, whereas the number in the G2/M phase increased (Fig. 5D). MUG-Chor1 untreated control cells showed a cell cycle distribution of G1: 74.8 ± 6.0, S: 2.9 ± 0.7, G2/M: 13.5 ± 2.0. Crizotinib treatment for 72 h changed the distribution to G1: 52.1 ± 10.7, S: 5.9 ± 2.2, G2/M: 23.1 ± 3.8. Cabozantinib treatment for 72 h changed the cell cycle distribution to G1: 41.4 ± 5.8, S: 4.4 ± 2.8, and G2/M: 29.8 ± 8.1. The significance was determined by comparing the individual cell cycle phases of the control with the cell cycle phases of the treated cells, which resulted in a significant of G1 (p = 0.0193), G2/M (p = 0.0328) treated with crizotinib and G1 (p = 0.033), G2/M (p = 0.046) treated with cabozantinib. U-CH2 untreated control cells showed a cell cycle distribution of G1: 38.07 ± 3.22, S: 8.83 ± 4.07, and G2/M: 31.93 ± 2.41. Crizotinib treatment for 72 h changed the distribution to G1: 32.73 ± 6.11, S: 8.07 ± 4.8, and G2/M: 21.33 ± 2.0. Cabozantinib treatment for 72 h changed the distribution to G1: 26.87 ± 7.61, S: 9.93 ± 5.7, G2/M: 25.47 ± 6.86. A significant difference was observed between the untreated control cells and under treatment with crizotinib in the G2/M phase (p = 0.0046).

### Annexin V/PI apoptotic induction

**Figure 6:**
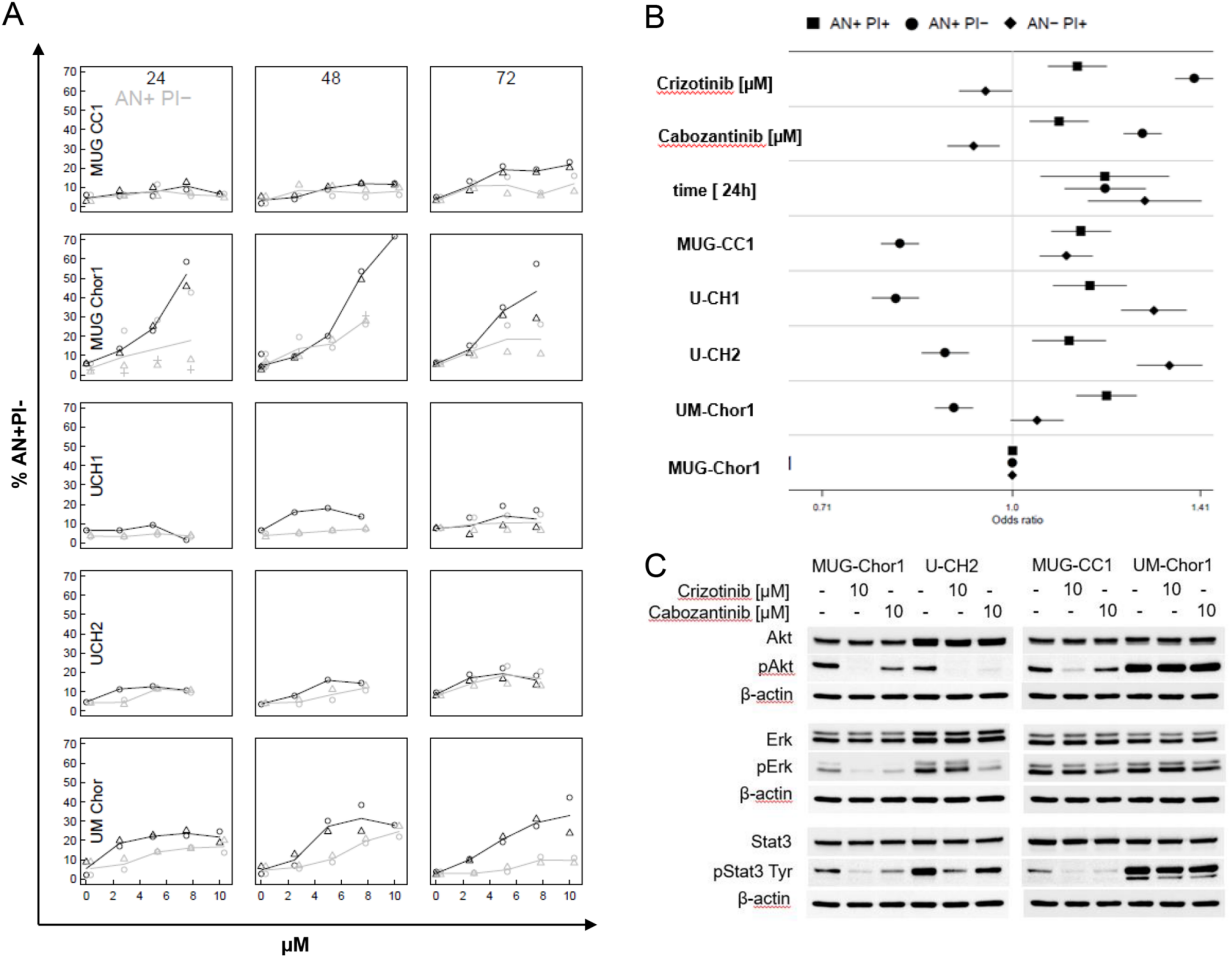
Apoptotic induction and MAPK regulation of the cMET inhibitors crizotinib and cabozantinib. (**A**) The vertical axis shows the percentages of the FACS “AN+PI−” for every cell line and every treatment. The horizontal axis depicts the dose of the cytotoxic agent in [µM]. Cell lines treated with crizotinib are shown in black, those treated with cabozantinib are shown in grey. A combination of color and symbol pertain to a single experiment, lines depict the mean of at most two replications. The symbols are slightly shifted against each other for visibility reasons. (**B**) Analysis of effect of the experimental factors dose, incubation time and cell line on AN+PI+, AN+PI− and AN−PI+ by a quasilogistic model. The ratio of the odds to observe each outcome is given for a concentration change of one µM, for an increase of incubation time by 24 h and for each cell line with respect to the reference cell line. Each symbol with line segment marks an odds ratio with 95%-confidence limit.

In order to investigate the apoptotic potential of the cMET inhibitors studied, we performed the Annexin V/PI FACS analysis. Figure 6A shows the dependence of the proportion of AN+PI− on the dose of the cytotoxic agent in [µM]. The results of the linear quasilogistic model were presented for the dependence of each of three cell fractions on cytotoxic agent concentration, incubation time and cell line, using MUG-Chor1 as a reference (ratio 1) (Fig. 6B). All three fractions increase with prolonged incubation time. AN−PI+ was inhibited by both crizotinib and cabozantinib. In contrast, the AN+PI− fraction increased due to cytotoxic agent; this increase was about three times stronger than the increase of AN+PI+. The AN+PI content showed the highest values in MUG-Chor1, whereas for AN+PI+ and AN−PI+ the opposite relationship could be noticed.

### Evaluation of downstream pathways after crizotinib and cabozantinib treatment

The cellular response to crizotinib and cabozantinib was assessed by measuring phosphorylation of the mitogen-activated protein kinases (MAPK) Akt, Erk, and Stat (Fig. 6C) by protein expression analysis after exposing the cells to 10 µM crizotinib respectively cabozantinib for 24 h. Both, crizotinib and cabozantinib, influenced the downstream pathways of cMET in chordoma cell lines. Crizotinib resulted in a significant decrease in Akt phosphorylation in the sacral cell lines MUG-Chor1 and U-CH2, as well as in MUG-CC1. The clival cell line UM-Chor1 showed no change in the phosphorylation pattern. Erk phosphorylation was significantly reduced in the sacral cell lines, whereas the clival cell lines remained unchanged. Similar to Akt, treatment reduced Stat3 phosphorylation in MUG-Chor1, U-CH2, and MUG-CC1, but it did not affect UM-Chor1. In summary, upon stimulation with HGF, the cMET inhibitors crizotinib and cabozantinib had a significantly stronger effect on protein phosphorylation in sacral chordoma cell lines compared to clival ones.

## Discussion

In the pathogenesis of various epithelial tumors, receptor tyrosine kinases such as cMET or EGFR, have been implicated and molecular-targeted therapies are being administered^11,12^. HGF, the only known ligand for the cMET receptor, is mainly expressed in cells of mesenchymal origin, although some epithelial cancer cells appear to express both HGF and MET^13,14^.

To better understand the role of cMET in chordomas, we studied whether the *cMET* gene-locus was polysomic or amplified in two sacral and two clival chordoma cell lines via single cell COBRA FISH analysis and copy number profiling. Comparing the number of allele/chromosomes within these cell lines, MUG-CC1 presented with 33 chromosomes, which comprises a nearly-haploid (< 30 chromosomes) karyotype; this cell line showed monosomy of chromosome 7. In contrast, the other chordoma cell lines displayed a varying polysomy of chromosome 7. Thus, heterogeneity between the karyotypes of these cell lines existed, which particularly affected chromosome 7, on which both, the *cMET* and *HGF* genes, are located^15^. Comparing chordoma cell lines after HGF stimulation, U-CH2 showed the strongest phospho-cMET—which is the activated form of cMET-expression, and MUG-CC1 the lowest. We found that sacral chordoma cell lines showed a stronger migration towards an HGF-gradient compared to clival cell lines. Cell migration and chemotaxis towards an HGF gradient have been described previously in the chordoma cell line CCL3^16^.

Although the overall results for cMET inhibitors in phase III clinical trials in non-small cell lung cancer are disappointing, subgroups of patients with *MET* gene alterations have been shown to benefit from cMET inhibitors^17^.

Therefore, we investigated whether sacral and clival chordoma cells respond differently to a treatment with cMET inhibitors. The dual MET/ALK inhibitor crizotinib showed a dose-dependent reduction of viability in various breast cancer cell lines after 48 h of treatment, with an IC_50_ of 5–10 µM^18^, which is similar to the IC_50_ obtained in our HGF-stimulated, crizotinib-treated chordoma cells. Yakes et al. tested the effect of cabozantinib, a small molecule tyrosine kinase inhibitor, on the proliferation behavior of different tumor cell lines and achieved different effects with respect to cMET amplification and IC_50_ concentrations^19^. Cell lines with cMET amplifications presented an IC_50_ between 10 and 19 nmol/L, cells without cMET amplification only an IC_50_ of 1–5 µmol/L. Our viability data clearly showed that, after HGF-stimulation, sacral chordoma cell lines responded better to treatment with cMET inhibitors than clival cell lines. In accordance with these data, treatment with cMET inhibitors caused a reduction of cells in the G1 phase, resulting in G2/M arrest of the cell cycle. It is well known that the cyclin B1/CDK1 complex together with cyclin A/CDK2 promotes G2/M transition in the eukaryotic cell cycle^20^. Compared to control cells, the expression of cyclin B1 and its corresponding cyclin-dependent kinase CDK1 was clearly reduced in MUG-Chor1 and U-CH2 cells. Thus, the checkpoint for the transition between the cell cycle phases was not being formed and the cells were arrested. Aberrant HGF/cMET axis activation, which is closely related to cMET gene polysomy and amplification, promotes tumor development and progression by stimulating the MAPK and other signalling pathways^21,22^. Our data revealed that the sacral chordoma cell lines showed a significant reduction in phosphorylation of Akt, Erk, and Stat3 after treatment with cMET inhibitors. Remarkably, UM-Chor1 showed no changes in these phosporylation patterns.

## Conclusion

For a future treatment of chordoma patients the origin of the primary tumor seems to play a crucial role. A high grade of heterogeneity between individual cell lines was observed. cMET inhibitors effected the growth behavior and cMET phosphorylation state of chordoma cells in different ways. Based on our in vitro cell culture analyses, it can be concluded that eventual treatment with cMET inhibitors would be more appropriate in sacral patients than in clival patients. Further investigation into chordoma heterogeneity will allow for a more precise treatment of individual patients and will pave the way towards a personalized chordoma treatment management.

## Methods

The authors confirm that all used methods were performed in accordance with the relevant guidelines and regulations.

### Immunohistochemistry (IHC)

Patient data and histological specimens were collected from the databases of the Diagnostic and Research Institute of Pathology, Medical University of Graz. The study protocol and the informed consent of patients were approved by the ethics committee of the Medical University of Graz (vote #18-192ex06/07). The informed consent was obtained from all participants. Nine formalin-fixed paraffin-embedded chordoma specimens (5 clivus, 4 sacral) were analysed. IHC stains were conducted with antibodies against S-100, CK, Vimentin, EMA, and brachyury (all Dako, Glostrup, Denmark). Only brachyury-positive samples (nuclear expression), in combination with a characteristic morphology were included in the study. cMET staining was performed with the mouse monoclonal MET antibody on the Leica Bond-III detection platform, using the Bond Polymer Refine Detection system (Leica).

### Cell culture

The sacral chordoma cell lines MUG-Chor1^23^ and U-CH2 and the clival chordoma cell lines UM-Chor1 (kindly provided by Chordoma Foundation, Boston, MA) and MUG-CC1^24^ were cultured in IMDM/RPMI 4:1 (Life Technologies, Carlsbad, CA, USA) containing 10% fetal bovine serum (FBS; Biochrom AG, Berlin, Germany), 1% insulin, transferrin, sodium selenite (ITS; Life Technologies), 2 mM glutamine, and 1% penicillin/streptomycin (Pen/Strep; Life Technologies). Incubation was carried out at 37 °C in a humidified atmosphere of 5% CO_2_. All cell cultures were periodically checked for mycoplasma by PCR, cell line identification was performed by STR analyses (Power Plex 16 System (Promega, Madison, WI, USA).

### Cobra fish

Cells were harvested using the chemically induced chromosome condensation technique published^25^. Slides with metaphase chromosomes were hybridized using a multicolor FISH approach, known as COBRA-FISH. The 43-color FISH staining of every chromosome arm in a different color combination, digital imaging and analysis were performed as described^26^. Chromosomal breakpoints were assigned using inverted images counterstained with 4',6-diamidino-2-phenylindole (DAPI; Downers Grove, IL, USA) together with the information derived from the short- and long-arm specific hybridization during COBRA-FISH. Karyotypes were described according to ISCN 2009. Two-color Fluorescence In Situ Hybridization (FISH) was performed as previously described^27^. BAC clone (BACPAC Resources Center) RP11-95I10 was selected to analyze the cMET locus. As reference DNA of centromere 7 was used. RP11-95I10 and DNA of centromere 7 were labeled Cy3-dUTP and Fluorescein-12-dCTP respectively using a nick translation labeling reaction^28^.

### Copy number profiling

Genome wide copy number alterations (CNA) were established using shallow whole genome sequencing (sWGS). Shotgun libraries were prepared using the TruSeq DNA LT Sample preparation Kit (Illumina, San Diego, CA, USA) following the manufacturer´s instructions. Briefly, after fragmentation and concentrating the volume to 50 µl end repair, A-tailing and adapter ligation were performed following the manufacturer’s instructions. Libraries were quality checked on an Agilent Bioanalyzer using a DNA 7500 Chip (Agilent Technologies, Santa Clara, CA, USA) and quantified using qPCR with a commercially available PhiX library (Illumina) as a standard. Libraries were pooled equimolarily and sequenced on an Illumina MiSeq in a 150 bp single read run. Copy number profiling from sWGS data was performed using the ichorCNA algorithm applying 1 Mb bin, a probabilistic Hidden Markov Model (HMM) model for the estimation of tumor fraction, roughly equivalent to tumor purity from bulk tumor analyses. Focal amplifications were called from 50 kb bins according to the criteria published in Ulz et al. 2016^29^.

### Western blot analysis

For immunoblotting, whole cell protein extracts were prepared with RIPA lysis buffer including protease inhibitors, subjected to SDS-PAGE and blotted onto PVDF membrane (Roth, Karlsruhe, Germany). Primary antibodies against cMET/phospho-cMET, Akt/phospho-Akt, Erk/phosphor-Erk, and Stat3/phosphor-Stat3 were purchased from Cell Signaling Technology (Danvers, MA, USA) and β-actin from Sigma-Aldrich (Vienna, Austria). Blots were developed using horseradish peroxidase-conjugated secondary antibodies (Dako, Jena, Germany) at room temperature for 1 h and the SuperSignal West Pico Chemoluminescent Substrate (Thermo Scientific, Rockford, IL, USA), in accordance with the manufacturers’ protocol. All original membranes can be found in the supplementary data.

### xCELLigence migration analysis

Real-time monitoring of cell migration was performed using the xCELLigence system with the CIM-Plate 16 (OLS, Bremen, Germany). When cells migrated through the microporous membrane into the bottom chamber they contacted and adhered to the electronic sensors, resulting in an increase in impedance. The cell-index showed the impedance changes and cell migration were automatically and continuously recorded every 15 min for 30 h. Acquisition and analysis was performed with the RTCA 2.0 software (OLS).

### xMAP technology

Cells were starved (without FBS) overnight and treated with 10 ng/ml HGF for 1 h. After incubation supernatant was stored at -80 °C for further analysis. The epidermal growth factor (EGF), the vascular endothelial growth factor (VEGF), interleukin 6 (IL-6), and the matrix metalloproteinase 9 (MMP9) levels were determined using the commercially available Affymetrix eBioscience lot number (ln) 107311000 (eBender MedSystems, Vienna, Austria) on a Bioplex200 system (Biorad, Hercules, CA) in combination with Bio-Plex Manager software, version 4.1 (Bio-Rad Laboratories, Hercules, CA, USA), with 5-parametric curve fitting. Standard range and sensitivity for the respective cytokines: EGF (ln 95592000) 2.93–12000; VEGF-A (ln 107198005) 5.37–22000 pg/ml; HGF (ln 99618101): 10–41700 pg/ml; IL-6 (ln 102903000) 9.57–9800 pg/ml; MMP9 (ln 97975000) 1.06–4350 pg/ml. Complete growth medium was used as background control.

### Viability assay

CellTiterGlo cell proliferation assay (Promega) was performed according the manufacturer's instructions. Cells were pretreated with 10 ng/ml HGF for 1 h and treated with the selective cMET inhibitors crizotinib (PF-02341066) or cabozantinib (XL184) (Selleckchem, Houston, TX, USA) for 72 h.

### Cell cycle analysis

After incubation with 5 µM crizotinib or 5 µM cabozantinib for 72 h, cells were fixed with 70% ice cold ethanol for 10 min at 4 °C. After washing, the cell pellet was resuspended in propidium iodide (PI) staining buffer (50 μl/ml PI, RNAse A; Beckman Coulter, Brea, CA, USA). Cell cycle distribution was analyzed by FACSCalibur (BD Biosciences, San Diego, CA, USA) using ModFit software.

### Annexin V/PI apoptosis assay

The APC Annexin V Apoptosis Detection Kit (BioLegends, San Diego, CA, USA) was performed following the manufacturer’s instructions. Apoptotic cells were identified by the incubation of 1 × 10^5^ cells in 100 µl Annexin V Binding buffer containing 5 µl Annexin V-APC and 5 µl PI for 15 min at room temperature. Flow cytometry analysis was performed with BD FACS LSR II (BD Biosciences). The experimental design of Annexin evaluation comprised the factors cell line, time, dose and treatment. The measurements from FACS represent relative frequencies which are binomially distributed with mean between 0 and 1 and variance depending on the mean. Taking the odds pertaining to a cell type of a cell culture with dose zero at time zero and cell line MUG-Chor1 as a reference, any factor combination can be predicted by multiplying the odds ratios of the individual factors. The odds ratios of time and dose were exponentiated by time and dose, respectively. This logistic model was implemented as a generalized linear mixed model (via penalized quasi-likelihood with replication as random factor) using the R (www.r-project.org, Version 3.5.2) and the package MASS 7.3–51.1.

## Supplementary Information

Supplementary Information.
