# Supplementary material for: Higher cMET dependence of sacral compared to clival chordoma cells: contributing to a better understanding of cMET in chordoma
Source: Sci Rep. 2021 Jun 14;11:12466. doi: 10.1038/s41598-021-92018-0 (PMC8203686; doi:10.1038/s41598-021-92018-0)
Supplement: Supplementary file 1 — Supplementary Information. [file 41598_2021_92018_MOESM1_ESM.pdf]

Supplementary data – original membranes of western blot analysis

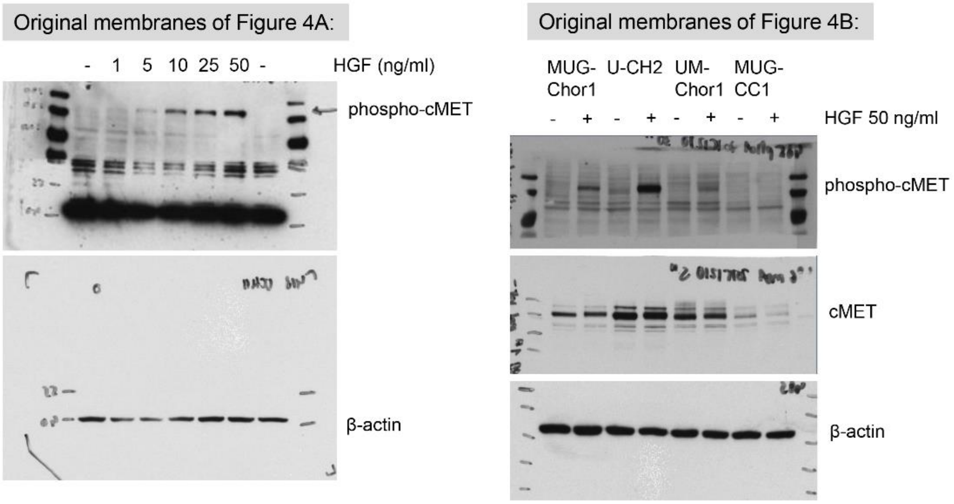

Figure S1. cMET phosphorylation and migration analysis after HGF stimulation. A) The concentration depending increase of phospho-cMET expression after the treatment with 0, 1, 5, 10, 25 and 50 ng/ml HGF; B) Protein expression of cMET and phospho-cMET after 50 ng/ml HGF treatment in all four chordoma cell lines. One representative blot out of three is shown. β-actin was used as loading control.

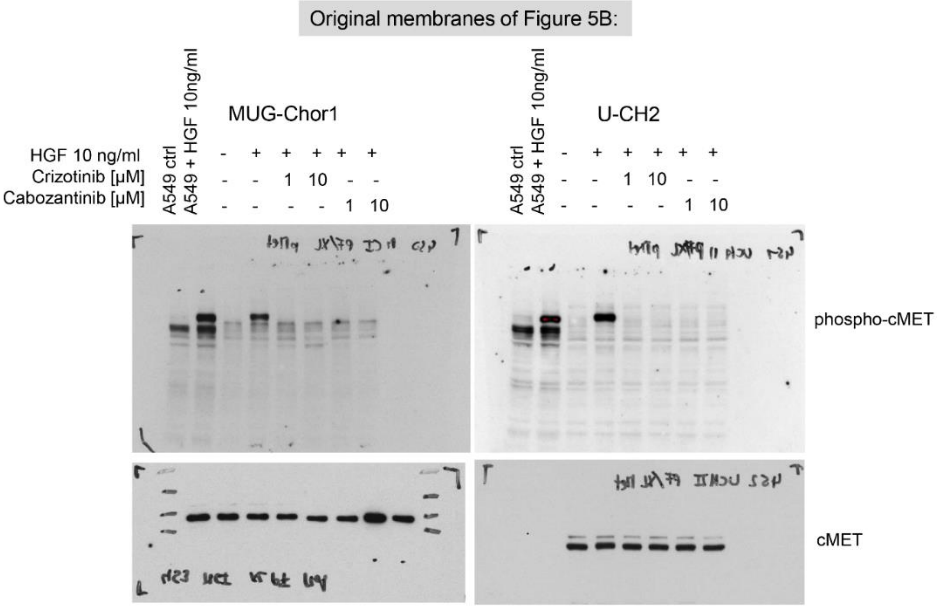

Figure S2. Influence of the cMET inhibitors crizotinib and cabozantinib on viability and cell cycle. B) Protein expression of cMET and phospho-cMET under the influence of 1 and 10 µM crizotinib and cabozantinib.

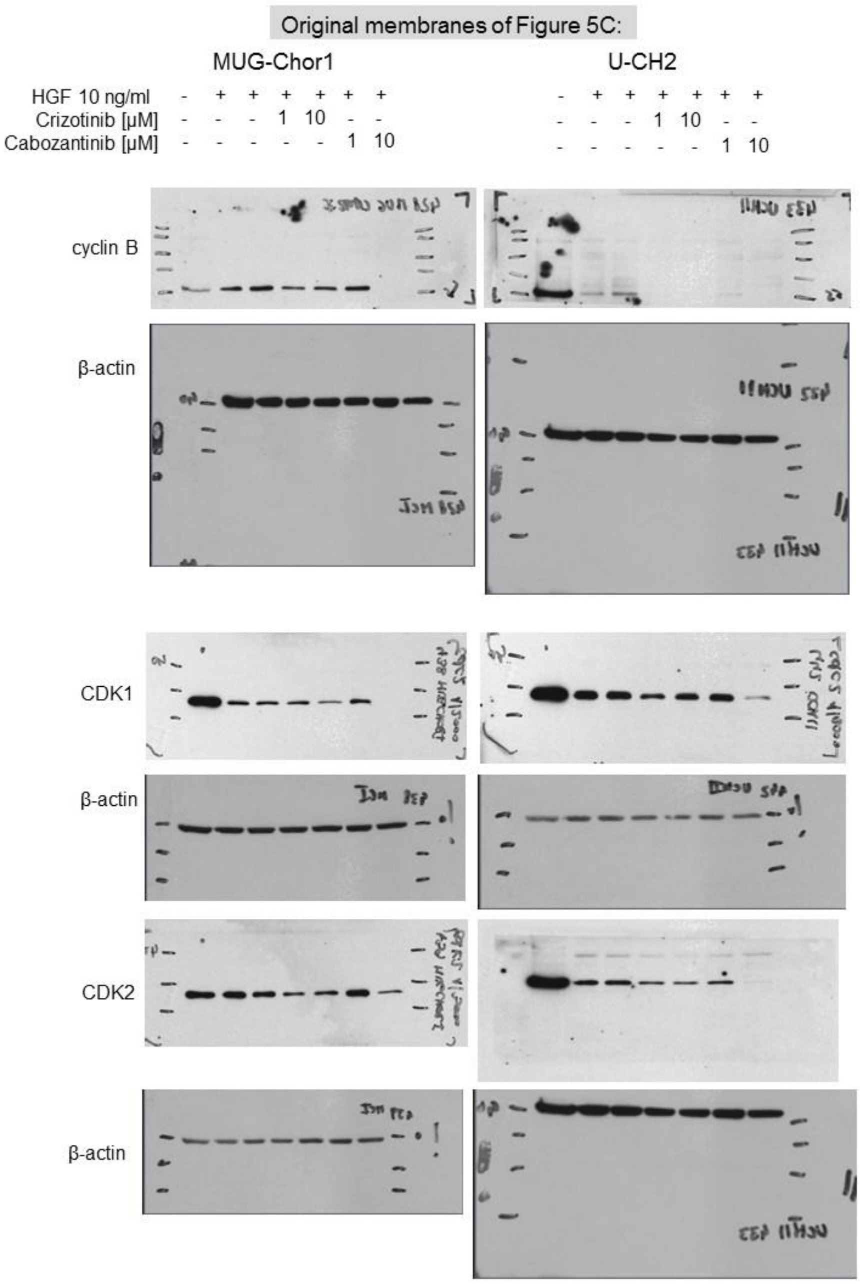

Figure S3. Influence of the cMET inhibitors crizotinib and cabozantinib on viability and cell cycle. C) Protein expression of the G2/M checkpoint proteins cyclin B, CDK1 and CDK2 were significantly reduced according to the G2/M arrest.

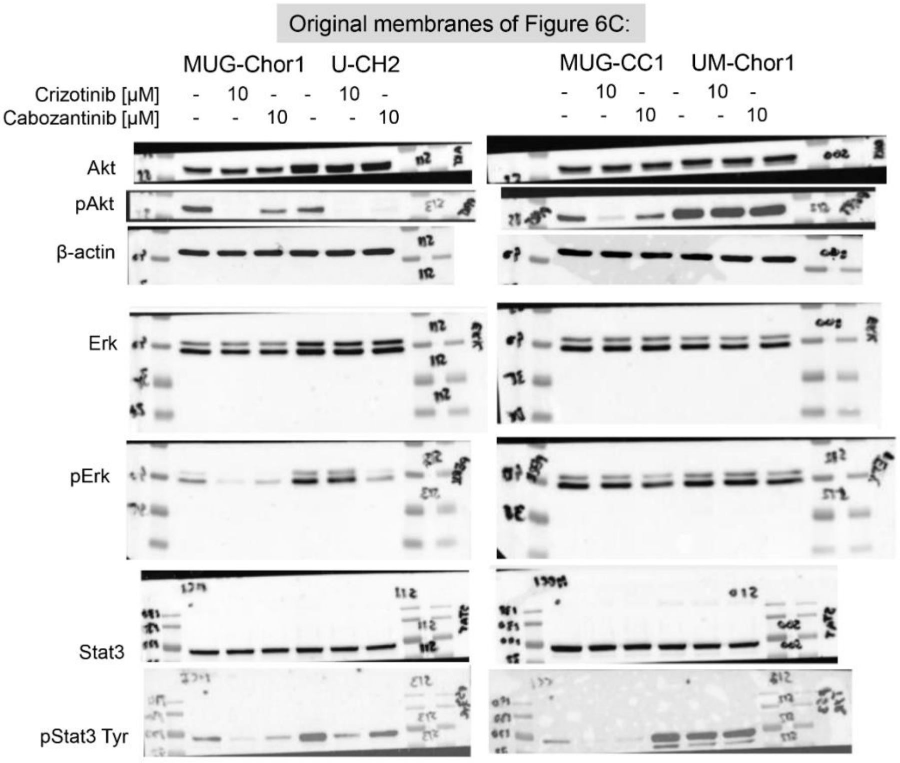

Figure S4. Apoptotic induction and MAPK regulation of the cMET inhibitors crizotinib and cabozantinib. C) Protein expression and phosphorylation levels of Akt, Erk, and Stat3 after treatment with 10 µM Crizotinib and Cabozantinib. The cMET inhibitors influenced individual the downstream pathways of cMET in chordoma cell lines.
